# Development and preliminary validation of a scale to measure self-efficacy in seeking mental health care (SE-SMHC)

**DOI:** 10.1186/s40064-015-1109-1

**Published:** 2015-07-11

**Authors:** Crystal Dea Moore, Casey Schofield, Dalena R M van Rooyen, Lena M C Andersson

**Affiliations:** Department of Social Work, Skidmore College, 815 N. Broadway, Saratoga Springs, NY 12866 USA; Department of Psychology, Skidmore College, 815 N. Broadway, Saratoga Springs, NY USA; Faculty of Health Sciences, School of Clinical Care Sciences, Nelson Mandela Metropolitan University, PO Box 77 000, Port Elizabeth, 6031 South Africa; Department of Social Work, University of Gothenburg, Sprängkullsgatan 23, PO Box 720, 405 30 Göteborg, Sweden

**Keywords:** Mental health, Help seeking, Self-efficacy

## Abstract

**Purpose:**

Globally, the prevalence of mental illness is on the rise, although few people with psychiatric disorders actually seek mental health care. One under-researched factor that may impact help-seeking behavior from health care professionals is self-efficacy. This research presents the development and validation of the Self-Efficacy to Seek Mental Health Care (SE-SMHC) scale, a nine item-self report measure. It was hypothesized that self-efficacy for seeking mental health care would be positively associated with higher rates of self-reported help-seeking behavior and higher rates of advising others in distress to access mental health treatment.

**Methods:**

A randomized population sample of 977 South Africans completed the SE-SMHC as part of a larger study on barriers to health care for mental illness. SE-SMHC data were subjected to principal component analysis, and data from the larger study were utilized to test the hypotheses.

**Results:**

Two latent factors emerged from the oblique rotation and accounted for 70% of the variance: SE-KNOW (confidence in one’s ability to know how to successfully interface with mental health care systems) and SE-COPE (confidence in one’s ability to cope with the consequences of seeking care). Cronbach alphas for both subscales were 0.87 and for the total scale score was 0.93. Both hypotheses were confirmed suggesting evidence of the scale’s validity.

**Conclusions:**

This data suggests that the SE-SMHC demonstrates good psychometric characteristics and may be a useful research tool and screening instrument for targeted interventions.

## Background

Globally, the prevalence of mental illness is on the rise. For example, the World Health Organization predicts that in 2020, depression will be the second most common disease contributing to disability adjusted life years, a measure of overall disease burden (World Health Organization 2001). Although effective evidence-based treatments exist for mental health problems (Gloaguen et al. 1998; Joffe et al. 1996), few people with psychiatric disorders actually seek mental health care (Alonso et al. 2004; Andersson et al. 2013; Bebbington et al. 2000; Forsell 2006; Hämäläinen et al. 2009; Kessler et al. 1994; Svensson et al. 2009).

Help-seeking can be defined as a process of actively seeking and using social relations, formal or informal, to receive help with personal problems. It involves both personal and interpersonal domains and the interaction between them (Rickwood et al. 2005). Different models have been developed to understand help-seeking behavior in general (Ajzen 1991; Andersen 1995) and for mental illness in particular (Biddle et al. 2007; Gulliver et al. 2012; Rickwood et al. 2005). Biddle and colleagues (Biddle et al. 2007) developed a dynamic model, the cycle of avoidance (COA), in order to understand reluctance to seek help for mental distress. In their qualitative interviews, they found that people were likely to wait to seek help until their symptoms were serious, compared to mental health clinicians who believed people should seek help earlier (before symptoms became unmanageable). Illness behavior was a long and circular process where individuals tried to negotiate and re-negotiate the worsening symptoms in order to understand and accept whether the illness was “normal” distress or “real” distress. Actually seeking help was avoided the longest. In short, participants struggled to successfully execute behavior that would promote wellness (i.e., treatment seeking).

A related model proposed by Rickwood et al. (2005) argues that help-seeking is a complex process that includes four crucial steps: awareness (and appraisal of the problem), expression (of symptoms and need for support), availability (of sources of help), and willingness (to seek out and disclose to sources). This work has revealed a number of factors that prevented seeking help, including a lack of emotional competence, help-negation (i.e., refusal to accept/access help that is available), and negative attitudes and beliefs about professional help. Factors that promoted help-seeking were supportive relationships, emotional competence, mental health literacy, positive experiences of previous care, and sense of mastery (Rickwood et al. 2005). Other epidemiological research has revealed reasons for not seeking care to be a belief that the problem will get better by itself (Meltzer et al. 2003), a desire to cope alone (Issakidis and Andrews 2002), feelings of shame (Forsell 2006), stigma (Link et al. 2001), and lack of social support (Forsell 2006). Together this work suggests that cognitive factors, such as understanding and appraisal of symptoms, are highly relevant to treatment-seeking, yet it is poorly understood how these factors undermine treatment seeking. Given the prevalence of mental illness coupled with the need to treat sufferers expeditiously, a better understanding of the cognitive and attitudinal dynamics of mental health help-seeking behavior is warranted in order to inform efforts to overcome barriers to care. This is particularly true given that the chance for recovery and increased well-being is increased with early detection and timely treatment.

Self-efficacy is an under-researched factor that may impact professional help-seeking behavior. Self-efficacy refers to people’s beliefs about their competence and abilities to activate personal resources that can help them exercise control over life events (Bandura 1997). Importantly, research on self-efficacy has demonstrated that people are more likely to engage in certain behaviors if they believe their efforts will be successful (Bandura 1997, 2006). Although there has been little work exploring the relationship between self-efficacy and treatment-seeking, Jackson et al. (2007) found evidence that one’s sense of self-efficacy may be a meaningful factor in promoting a healthy lifestyle in general. Further, increased perceived stigma about mental illness is associated with low self-efficacy (Kleim et al. 2008). As previously discussed, Rickwood et al. (2005) and Biddle et al.’s (2007) work suggests that help-seeking behavior is strongly influenced by cognitive appraisal. Negative appraisal of one’s ability to successfully access treatment and improve mental health symptoms (i.e., low self-efficacy for mental health treatment) may undermine appropriate treatment seeking. These preliminary findings suggest that self-efficacy—an individual characteristic amenable to change—is worthy of further investigation to better understand its role in help-seeking behavior for mental illness.

Self-efficacy beliefs impact a variety of domains relevant to help-seeking: emotional, cognitive, motivational, and behavioral outcomes (Bandura 1994). Specifically, help-seeking requires the emotional skills to acknowledge the problem and belief in the value of getting help, the cognitive capacity to know where and how to get help, and ultimately the motivation to engage in the appropriate behaviors to access care. Among other things, the value of being able to measure self-efficacy to seek mental health care is the potential it provides to identify individuals and communities that may particularly benefit from targeted public health campaigns and interventions. These may include knowledge-focused and confidence-enhancing trainings aimed at promoting appropriate help-seeking. Such programs are likely to be particularly impactful in regions and communities where there is a clear disconnect between the presence of psychiatric symptoms and access to psychiatric care.

One such area is the continent of Africa, particularly South Africa, where mental health care has historically been a relatively low priority behind other pressing public health burdens: HIV/AIDS, tuberculosis, high maternal and child mortality rate, non-communicable diseases, and violence, injury, and trauma (Motsoaledi 2013). In fact, “education of the public” in Africa has been noted as one of the important priority areas for mental health policy development (Gureje and Alem 2000). To this end, the Grand Challenges in Global Mental Health Initiative has recently highlighted the importance of understanding disparities in treatments and outcomes for mental health issues in low income countries (Collins et al. 2011). Previous research in South Africa suggests that as much as 30% of the population suffers from a mental illness in their lifetime (Herman et al. 2009), but most never access treatment (Andersson et al. 2013; Seedat et al. 2008, 2009). Initial work addressing this discrepancy has indicated that low treatment utilization in South Africa may be explained in large part by attitudinal barriers associated with self-efficacy (e.g., stigma, appraisal of one’s ability to access treatment, assessment of one’s capacity to effectively communicate about the illness) (Andersson et al. 2013; Bruwer et al. 2001). Again, despite its relevance to such attitudinal and cognitive barriers, the role of self-efficacy has received limited attention in characterizing barriers to mental health help-seeking.

The goal of the current study is to develop and validate a scale measuring self-efficacy for mental health care, the self-efficacy to seek mental health care scale (SE-SMHC). This study is part of a larger investigation of barriers to care among persons with mental illness in primarily poor areas in South Africa (Andersson et al. 2013). Using a subset of the data from the larger study, the psychometric properties of this nine-item measure were preliminarily evaluated through principal component analysis, reliability analyses, and known-groups validity. In terms of the latter, differences on the SE-SMHC between participants who did (versus did not) seek help in the past when they were emotionally distressed were explored given that such treatment seeking required that the individual demonstrated the competence to take control of one’s own mental health care (i.e., provides evidence for self-efficacy in this domain). Therefore, it was hypothesized that individuals with a history of help-seeking would have higher scores on the SE-SMHC than those who did not (higher scores indicate increased self-efficacy). In addition, given that one would expect individuals who reported that they would advise another (emotionally troubled) person to seek mental health care would have a stronger sense of competence about their ability to provide mental health advice (and understanding of how to initiate such help-seeking behavior) compared to individuals who would not provide such advice, these group differences were also explored. It was hypothesized that individuals who indicated that they would advise someone in distress to access mental health treatment would have higher SE-SMHC scores than those who would not provide such advice. Given that self-efficacy beliefs are likely to influence behavioral outcomes, these hypotheses are intended to demonstrate known-groups validity. As such, this study represents an initial step in characterizing a potentially important component of the well-documented attitudinal barriers to appropriate mental health help-seeking in a large population of young adults in South Africa. Given that self-efficacy is both amenable to change and likely integral to the help-seeking process, and no measure of the construct could be identified, the current study is designed to introduce and validate the SE-SMHC to fill this gap in the literature.

## Results

To explore the psychometric qualities of the SE-SMHC, the data were subjected to principal component analysis, and t tests to explore the scale’s validity.

### Factor structure of the SE-SMHC

Principal component analysis was used to explore the SE-SMHC’s latent factor structure. Examination of the correlation matrix revealed significant correlations among the scale items with no correlations over 0.90 suggesting that singularity was not an issue for any of the items (correlations ranged from 0.39 to 0.71). The Kaiser–Meyer–Olkin measure of sampling adequacy was 0.90 and Bartlett’s Test of Sphericity revealed that factor analysis was appropriate for these data: χ^2^ (36, *N* = 944) = 5049.94, *p* < 0.001. The data were subjected to an oblique rotation which provided the most statistically and theoretically sound solution. Two items which addressed one’s confidence in the ability to pay for transportation and pay for mental health services were dropped from the scale as many of the resulting factor loadings among the 11 items were below 0.40, and the pattern of the loadings did not make theoretical sense. In fact, payment for transportation and mental health services is unlikely to be a universal concern across groups in different settings, so in addition to the problematic factor loadings, this provided an additional theoretical reason to drop these items from scale (in order for the content to be applicable to as many groups as possible). Examination of the scree plot and associated eigenvalues for the final nine items revealed two latent variables. Results of the final model are presented below.

The eigenvalues and the variance uniquely explained by each factor were: Factor 1, 5.19, 58%; and for factor 2, 1.04, 12%, for a total of 70% of the variance accounted for by the two factors. Cronbach’s alpha was 0.93 for the total scale score (SE-TOTAL), and for both factors 1 and 2, the value was 0.87. Factor loadings for factor 1 ranged from 0.69 to 0.88, and for factor 2, −0.74 to −0.88. Factor 1 captured self-efficacy in finding information about how to successfully interface with mental health care systems (Confidence in Knowledge subscale—SE-KNOW), and Factor 2 addressed participants’ confidence in their ability to cope with consequences of seeking care (Confidence in Coping subscale—SE-COPE). The correlation between the two subscales was significant, *r* = 0.67, *p* < 0.001. See Table 1 for factor loadings and descriptive statistics for the items. This table provides the content SE-SMHC in its entirety as well instructions for scoring.

**Table 1:** Factor loadings, descriptive statistics, and instructions for administering and scoring the SE-SMHC

Item	Communalities	Factor 1 loadings	Factor 2 loadings	Item mean	Item standard deviation
1. Find a place to get mental health treatment	0.59	0.70		7.31	3.05
2. Get transportation to a mental health care service	0.64	0.83		7.37	3.06
3. Clearly tell the staff what is troubling me	0.71	0.88		7.84	2.62
4. Understand the information given to me by the staff	0.74	0.88		8.08	2.36
5. Be able to follow the treatment recommendations made by the staff	0.68	0.69		8.25	2.24
6. Cope well with the consequences of seeking care (for example, treatments, tests, hospitalizations)	0.69		−0.74	7.43	2.69
7. Cope well with my family and friends reactions to me seeking mental health treatment	0.75		−0.85	7.28	2.78
8. Cope well with the attitudes that the staff may have towards me	0.69		−0.86	6.73	2.98
9. Overcome any embarrassment I may have about seeking mental health treatment	0.76		−0.88	7.03	2.94

Factor loadings <0.20 are not reported. To administer the scale, the nine items should be preceded by these instructions: Below are several statements about your confidence in your ability to seek mental health care if you ever needed it. For each statement, rate how confident you are from 1 = no confidence to 10 = complete confidence in your ability to do each behavior. There are no right or wrong answers. We are interested in how you see yourself and your own abilities. To score the subscales, add up the ratings for SE-KNOW subscale (SE-KNOW scores will range from 5 to 50) and SE-COPE subscale separately (SE-COPE scores will range from 4 to 40). For the total scale score, add up the ratings for all 9 items (SE-TOTAL scores will range from 9 to 90).

### Validity of the SE-SMHC

Relevant variables from the structured interview schedule were selected to explore their relationship with the SE-SMHC. In terms of demographics, a series of independent group t tests and one-way ANOVAs were calculated for the two subscales and total SE-SMHC score to determine if there were significant differences between men and women as well as younger and older participants. The tests for gender and age were not significant, but there was a trend suggesting that women tended to have higher scores on the SE-COPE subscale and total scale (SE-TOTAL) score (p = 0.054 and p = 0.076, respectively). In terms of education, one-way ANOVAs revealed that there were significant differences for educational groups (completed university/college, completed high school, completed primary school, did not complete primary school) for both subscales and the total score, suggesting that increased education was associated with higher self-efficacy scores: SE-KNOW, F (3, 973) = 16.07, p < 0.001; SE-COPE F (3, 973) = 6.16, p < 0.001; and SE-TOTAL F (3, 973) = 10.89, p < 0.001.

In order to explore the validity of the SE-SMHC and to test the study’s hypotheses, items from the structured interview schedule that reflected reported the participants’ own help-seeking behavior and willingness to encourage help-seeking behavior in others were examined. Participants were asked if at some point they had felt “so emotionally troubled that they felt a need to seek help” (814 responded “yes”). They were then asked if they did “seek help from relatives or other trusted people.” As predicted, there were significant differences on both subscales and total score such that those who did seek help (n = 294) scored significantly higher than those who did not seek help (n = 520): SE-KNOW, t = −4.46 (812); SE-COPE t = −3.39 (812); and for SE-TOTAL t = −4.34 (812), all *p*s < 0.001. Known-groups validity was further established by evaluating whether participants who indicated “yes” to the question, “Did you seek care from any health care staff when you felt emotionally troubled?” (n = 294) had higher scores on both subscales and the total score of the SE-SMHC than those who answered no (n = 520): SE-KNOW, t = −4.76 (730); SE-COPE t = −3.60 (720); and for SE-TOTAL t = −4.67 (743), all *p*s < 0.001. Similarly, individuals who reported that they would advise another person to seek health care if the individual were emotionally troubled (n = 937) scored significantly higher on both subscales and the total score of the SE-SMHC than those who would not (n = 40): SE-KNOW, t = −4.09 (42); SE-COPE t = −3.07 (42); and for SE-TOTAL t = −4.09 (42), all *p*s < 0.005.

## Discussion

This data suggests that the nine-item SE-SMHC demonstrates good psychometric characteristics. The latent factor structure reveals two separable constructs that are theoretically sound. The first subscale captures respondents’ confidence in their ability to successfully interface with the health care system, including confidence in knowing how to access health care and in the ability to communicate with professionals within this system. The second subscale captures participants’ confidence in their ability to cope with both social and intrapersonal consequences of seeking care. Both subscales and the total scale revealed strong internal consistency, and analyses also provide preliminary evidence supporting the validity of the SE-SMHC.

Consistent with the study’s initial hypotheses, individuals who reported a history of seeking help for emotional distress reported higher levels of self-efficacy for seeking mental health care (both from trusted people in the community as well as from health care providers) compared to individuals who did not seek care when emotionally distressed. In addition, individuals who indicated that they would advise someone else to seek care also reported significantly higher levels of self-efficacy for mental health care compared to those who indicated they would not recommend seeking care for emotional distress. These results are consistent with previous work suggesting that self-efficacy is associated with improved health-related behaviors (Marks et al. 2005) as well as theoretical claims that higher levels of domain specific self-efficacy are associated with successful behavioral outcomes. Such findings are particularly encouraging because previous research has demonstrated that self-efficacy is a malleable construct, and that improvement in self-efficacy is related to positive health-related behavior change (e.g. Allison and Keller 2004; Luszczynska et al. 2006; Tsay 2003). Thus, the ability to effectively measure self-efficacy as it relates to mental health help-seeking is an important step towards characterizing the role this construct may play in improving access to mental health treatment.

Such a measure is most likely to be useful by workers in primary health care, public health, and social work in combination with other measures in seeking to identify those who would most benefit from interventions designed to enhance attitudes toward and improve access to mental health care. To this end, targeted media and public education campaigns may be developed to improve mental health help-seeking behaviors in these high risk groups that promote confidence in one’s knowledge and coping capacities. Of note, there has been encouraging evidence from community awareness public education campaigns in Australia targeting knowledge about mental health problems, suggesting that public education campaigns can produce change in targeted outcomes around mental health (Jorm et al. 2006). Efforts to promote access to mental health treatment may benefit from addressing self-efficacy for treatment seeking and are particularly important given that help-seeking for mental health issues typically occur only after significant delay (e.g., the delay to treatment is typically approximately a decade or more for depression and anxiety disorders (Wang et al. 2005). Data from this sample indicate that of those who indicated that they had been so emotionally troubled they felt they should seek help, 64% did not seek the needed assistance. Delays in seeking care are meaningful in that they not only represent longer episodes of unnecessary suffering, but also that delaying treatment is associated with worse outcomes for the majority of mental health problems (Post and Weiss 1998; Wang et al. 2005).

While this study has several strengths, it has limitations as well. This psychometric evaluation was conducted with a population-based sample of South African adults; however, the sample lacks diversity in terms of age (all participants were 40 years old or younger), geography, and ethnicity (84% Black). Given that there were significant differences for educational groups and a trend for one subscale indicating that gender may influence scores, other studies should attend to these individual difference variables to further illuminate factors that are associated with self-efficacy to seek mental health care. Future research should also include confirmatory factor analysis with other populations to determine if the factor structure that emerged from this exploratory analysis is consistent across groups with differing demographic characteristics. Next steps should also include an analysis of measurement invariance as well as cross-cultural invariance.

Further, because the current study did not include a measure of general self-efficacy, the results cannot speak directly to the relationship between the domain-specific self-efficacy for seeking mental health care and the more general construct of self-efficacy. Future investigations that explore the relationships between these constructs will be particularly important given that previous work suggests that general self-efficacy may have an inverse relationship with help-seeking behavior. Specifically, in a rural population, high general self-efficacy has been associated with *low* levels of help-seeking, presumably related to the agrarian values of assuming self-responsibility for health problems (Judd et al. 2006). The results of the current study may reflect a unique relationship between help-seeking and the domain-specific self-efficacy for mental health treatment, or meaningful differences between the study sample and the rural population used in previous research.

## Conclusions

This study fills a gap in the literature by introducing a short, easy-to-administer scale with promising psychometric qualities that measures self-efficacy to seek mental health care. The scale was validated on a predominately poor population, and provides insight into how self-efficacy to seek mental health care may explain the low rates of help-seeking behavior among this group. Given no such instrument exists, this scale is valuable in order to better understand how factors that facilitate or obstruct help-seeking for mental health care operate. Furthermore, it may promote the evaluation of interventions to improve self-efficacy for treatment seeking. Researchers using the scale should perform confirmatory factor analysis on their samples to ensure the factor structure that results from this analysis holds up for groups with differing demographic characteristics.

## Methods

### Sample selection and data collection

The survey was performed in the Nelson Mandela Bay Metropolitan Municipality of the Port Elizabeth area (1,005,780 inhabitants) and the Kirkwood area in the Cacadu District Municipality (412,000 inhabitants) in Eastern Cape, South Africa during March and July 2012. The research focused on this area to help illuminate the mental health needs of the inhabitants of this locale in South Africa and to include participants from both urban and rural areas. A total of 1,000 participants aged 18–40 years old were randomly sampled from the population for the study. This age range was chosen because mental illness often emerges during adolescence and young adulthood and, if not treated, may affect future health and socioeconomic status (Patel et al. 2007).

A complex multi-staged sampling process was conducted to provide a random population-based sample that reflected the regional and racial (Black, White, Coloured) distribution of South Africa. The selection of participants was conducted in stages and in different levels (e.g. region, district, household) using selection probabilities for different stages and with different strata. In basic terms, enumeration areas (EAs) were identified, each with approximately 500 people. The EAs were used for the sampling and based on regional distribution and race. The EAs were divided into three large racially dominant geographic areas, and selected EAs resulted in a representative distribution of the demographic race profile of South Africa. Ten households were randomly selected in each EA and household members randomly selected using the Kish method (Kish 1949) resulting in a random population-based sample. Hawth’s Analysis Tools for ArcGIS (Spatialecology, 2014) were used in conjunction with population statistics estimates by a professional statistical firm for determining the sample. More details describing the method is presented in Andersson et al. (2013).

Participants were given information about the study’s purpose, informed that they could withdraw from the interview at any time, and asked to complete a written consent. An extensive semi-structured questionnaire was developed in English including socioeconomic, demographic, health and perceived barriers to care questions and questions concerning self-efficacy related to help-seeking for mental illness (SE-SMHC). In the Eastern Cape Province three large language groups exist: English, Afrikaans and Xhosa. Trained interviewers conducted face-to-face interviews in the participants’ preferred language. (Information about translation is presented in the section on Measures.) Each interview took approximately 30–40 min and was performed in a private place chosen by the respondent. The study had a response rate of 97.7%, yielding a total of 977 participants. Demographic characteristics are presented in Table 2. Of note, the majority of study participants were unemployed (61%). This rate of unemployment is consistent with another South African study which found an unemployment rate of 65% (Bruwer et al. 2001). The majority of the respondents lived in poor areas where high rates mental illness exist (Andersson et al. 2013).

**Table 2:** Characteristics of the 977 participants (52% men, 48% women)

	N	%
Place or residence		
Port Elizabeth urban/semi urban area	832	85
Kirkwood: rural area	145	15
Age		
30–40	346	35
18–29	631	65
Ethnicity		
Black	821	84
Colored	86	9
White	59	6
Asian	9	0.9
Don’t want to respond	2	0.2
Marital status		
Married/cohabiting	158	16
Single, not in a relationship, widowed	819	84
Highest education		
Completed tertiary education	99	10
Completed high school	479	49
Completed primary school + uncompleted	399	41
Employment status		
Employed, full, part-time, self-employed	307	31
Unemployed and casual work	670	69
Weekly household income		
1001: >4,000 RAND	219	23
None: 1,000 RAND	588	60
Don’t want to disclose	170	17

### Measures

The SE-SMHC was part of a survey including both previously validated scales and new items developed specifically for the population-based study that examined barriers to health care in a sample of individuals in the Eastern Cape Province of South Africa. The questionnaire was translated and back translated from English to Xhosa and from English to Afrikaans by translators to accommodate the respondents’ preferred language. No cross-cultural equivalence testing was performed. However, a pilot study was conducted and ambiguities on language and other inconsistencies were clarified.

The focus of this analysis is to explore the psychometric qualities of the *Self Efficacy for Seeking Mental Health Care* (SE-SMHC) measure, a newly developed nine-item instrument. The scale was constructed considering the recommendations made by Bandura (2006) regarding the construction of self-efficacy scales. For example, Bandura suggests items should accurately reflect the construct of self-efficacy (i.e. perceived capability), and “…be phrased in terms of *can do* rather than *will do* [italics in the original]” (Bandura 2006). This is reflected in the SE-SMHC as the stem for all items reads, “How confident are you in your ability to…” Bandura also suggests that a good efficacy scale should accurately reflect the domain of functioning that is being assessed, and the content of the items be directly related to factors that determine quality of functioning in the specific self-efficacy domain. The content of the items was developed based on a review of previous research related to barriers to mental health care that addressed access (Fortney et al. 1993; Issakidis and Andrews 2002; Meltzer et al. 2003) and mental health literacy (Jorm et al. 1997), particularly knowledge, insight, and ability to follow through with treatment recommendations; as well as psychosocial factors, including stigma (Komiti et al. 2006; Link and Phelan 2001). A list of 11 items was developed that reflected the themes from the literature previously cited and attempts were made to make the scale as short as possible to increase ease of administration. Based on their clinical experience and the literature review, the authors judged these items as accurately capturing the domain of self-efficacy to seek mental health treatment. These items were then reviewed by an expert in mental health care (PhD-level licensed clinical social worker), and the scale was piloted with three lay persons. Small changes were made to the wording of items to enhance clarity. As a result of the principal component analysis, two items were deleted which resulted in the most statistically robust and theoretically sound pattern of factor loadings, and the final scale consists of nine items. In accordance with Bandura’s (2006) suggestion that self-efficacy measures should use a 10 or 100-point scale (with labelled anchors at the extreme) to rate the strength of belief such that scale sensitivity and reliability are enhanced, the final scale utilizes a 10-point Likert response scale with the anchors of 1 = no confidence to 10 = complete confidence in the respondent’s ability to do each behavior.
